# Investigating the Utility of Oblique Tree-Based Ensembles for the Classification of Hyperspectral Data

**DOI:** 10.3390/s16111918

**Published:** 2016-11-15

**Authors:** Nitesh Poona, Adriaan van Niekerk, Riyad Ismail

**Affiliations:** 1 Department of Geography and Environmental Studies, Stellenbosch University, Stellenbosch 7602, South Africa; avn@sun.ac.za (A.v.N.); riyadi@sun.ac.za (R.I.); 2 School of Plant Biology, University of Western Australia, 35 Stirling Hwy, Crawley, Perth, WA 6009, Australia

**Keywords:** hyperspectral data, oblique tree-based ensembles, spectral resampling, *Pinus radiata*

## Abstract

Ensemble classifiers are being widely used for the classification of spectroscopic data. In this regard, the random forest (RF) ensemble has been successfully applied in an array of applications, and has proven to be robust in handling high dimensional data. More recently, several variants of the traditional RF algorithm including rotation forest (rotF) and oblique random forest (oRF) have been applied to classifying high dimensional data. In this study we compare the traditional RF, rotF, and oRF (using three different splitting rules, i.e., ridge regression, partial least squares, and support vector machine) for the classification of healthy and infected *Pinus radiata* seedlings using high dimensional spectroscopic data. We further test the robustness of these five ensemble classifiers to reduced spectral resolution by spectral resampling (binning) of the original spectral bands. The results showed that the three oblique random forest ensembles outperformed both the traditional RF and rotF ensembles. Additionally, the rotF ensemble proved to be the least robust of the five ensembles tested. Spectral resampling of the original bands provided mixed results. Nevertheless, the results demonstrate that using spectral resampled bands is a promising approach to classifying asymptomatic stress in *Pinus radiata* seedlings.

## 1. Introduction

Hyperspectral data is characterized by a large number of contiguous bands, ranging from the visible through to the shortwave infrared portion of the electromagnetic spectrum [1]. For the analysis of plant stress, the high spectral resolution allows for the detection and quantification of a plant’s physiological response to stress [2]. This physiological response is exhibited as subtle variations in a plant’s spectral response, providing the basis for developing stress detection models [3,4]. Hyperspectral data subsequently provides the opportunity to readily monitor pest and disease stress in agricultural crops and forestry, as demonstrated by [3,4,5,6] and others.

The utility of hyperspectral data, especially spectroscopic data, is well established in the remote sensing domain for pest and disease detection. For example, the visible-near infrared (VNIR) spectrum has been particularly useful for the detection of stress in agricultural crops. Chávez et al. [7], used the 350 nm to 850 nm spectral range to detect bacterial wilt infection caused by *Ralstonia solanacearum* in potato crops. Similarly, [8] employed leaf and canopy VNIR reflectance data (325 nm to 1075 nm) to detect damage in rice crops caused by *Cnaphalocrocis medinalis*. Within a forestry context, [9] used the complete spectral range (350 nm to 2500 nm) to model degradation in *Avicennia germinans* and *Rhizophora mangle*. The VNIR and shortwave infra-red (SWIR) range was also utilized by [3] for modelling asymptomatic *Fusarium circinatum* stress in *Pinus radiata* seedlings. However, spectroscopic data is highly correlated and there is an a priori assumption that most of the bands will be redundant with only a few key bands producing the best result (see for example [3,4]). Additionally, the limited number of samples (*n*) available coupled with the large number of bands (*p*) presents a statistical challenge [10,11].

The random forest (RF) algorithm [12] is particularly well suited for addressing the challenges posed by high dimensional spectral data (see for example studies by [3,4,5,13]). Random forest reduces bias (systematic error term independent of the training sample) as well as variance (error due to variability associated with the training sample) by creating unpruned trees thus keeping bias low, and uses randomization for controlling the diversity between trees in the ensemble [14]. Randomization is introduced into the ensemble by creating trees using bootstrap aggregation with replacement of samples, as well as for selecting variables that will be used for node splitting [15].

However, RF suffers from two primary limitations. First, tree construction is based on a single feature being selected for node-splitting. Such trees may be inefficient in dealing with feature dependencies likely inherent in high dimensional spectral data [14]. Second, the majority of current implementations of the RF algorithm utilizes orthogonal splits based on univariate decision trees (DT). According to [16] the decision boundary generated from orthogonal splits of univariate trees may not be optimal for handling high dimensional spectral data. The argument is that a staircase or box-like decision boundary generated by univariate splits may not be optimal for highly correlated data, such as spectroscopic data, because the data may appear inseparable when their marginal distributions are evaluated [16]. Building on the initial recommendation of [12], [16] advocated the creation of multivariate DT by applying a supervised model to learn the splitting rule that results in oblique boundaries rather than the geometrical constrained boundary of orthogonal trees. To date, the only published remote sensing study that employed oblique RF (oRF) was by [17] for land cover and land use mapping.

Research by [14] on 15 high dimensional datasets showed that oRF using a support vector machine (SVM) as the node splitting model (oRFsvm) produced higher classification accuracies compared with using the traditional RF and SVM. Overall findings showed that using the oRFsvm model resulted in an improvement in the mean classification accuracy of 3.57% and 6.35% when compared with the traditional RF and SVM classifiers respectively. Similarly, [16] compared the oblique version of RF together with seven other classifiers, including RF and SVM, for the classification of high dimensional spectral data. Overall results showed that oRF outperformed all classifiers, with oRF using ridge regression providing the best results.

A related oblique tree-based ensemble approach is rotation forest (rotF) [18]. Unlike oRF that uses supervised models to determine the optimal split direction, rotF applies principal components analysis (PCA) on bootstrap samples to derive the optimal rotation of the axes for node splitting. Rotation forest encourages diversity in the model through random subset selection and using PCA for feature selection. High accuracy is sought through preserving the discriminatory information of the training data by retaining all the principal components [18]. Within a remote sensing context, [19] applied rotF for the classification of multispectral WorldView-2 data highlighting its superior performance over the RF, SVM, and nearest neighbor algorithms. Du et al. [20] also found that rotF outperformed RF and SVM when applied to the classification of fully polarimetric synthetic aperture radar imagery. Two studies [21,22] applied rotF for the classification of Airborne Visible Infrared Imaging Spectrometer (AVIRIS), Reflective Optics System Imaging Spectrographic (ROSIS), and Digital Airborne Imaging Spectrometer (DAIS) data. Results showed that rotF outperformed all classifiers including RF and SVM.

Several studies have successfully applied hyperspectral data for asymptomatic stress detection. For example, [23] used multi-temporal spectroscopic data with LDA to detect sugarcane yellow leaf in sugarcane plantations, caused by *Polerovirus*. Two studies [24,25] applied high resolution hyperspectral imagery with LDA and SVM classifiers to discriminate verticillium wilt severity in olive plantations, caused by *Verticillium dahliae*. De Castro et al. [26] used spectroscopic data with ANOVA and neural network classifiers to model laurel wilt severity in avocado crops caused by *Raffaelea lauricola*. Only two studies [3,4] have previously investigated the use of hyperspectral data for modelling *F. circinatum* stress in *P. radiata*, and discriminating healthy and stressed seedlings.

A previous study by [3] successfully demonstrated the use of the RF ensemble for modelling asymptomatic stress in *Pinus radiata* seedlings. The authors applied RF with the Boruta algorithm [27,28] for waveband selection and classification of healthy, infected, and damaged *P. radiata* seedlings. Results of their study indicated that hyperspectral data can successfully discriminate *F. circinatum* stress (discrimination of healthy and infected seedlings was achieved with accuracies above 80%). The authors further demonstrated that selected bands can potentially be used to discriminate stress with improved accuracy. Another study [4] confirmed the findings of [3] and additionally showed that a combination of selected bands could be used for modelling *F. circinatum* stress in *P. radiata* and *P. patula* seedlings.

It is within this context that we evaluated the utility of the RF, oRF, and rotF ensembles for the classification of hyperspectral data. The study was undertaken as a series of experiments. We first tested the five ensemble classifiers, i.e., RF, rotF, and oRF (with ridge regression, partial least squares, and SVM as the node splitting models) using all hyperspectral bands (*n* = 1769). We then evaluated the effect of decreasing the spectral resolution on the classification performance of the five ensemble classifiers. More specifically, we applied the RF, rotation forest, and oRF ensemble classifiers to modelling asymptomatic stress in *P. radiata* seedlings associated with *Fusarium circinatum* infection.

## 2. Materials and Methods

### 2.1. Fusarium Circinatum

*F. circinatum* (synonym *Gibberella circinata*) [29] is a fungal plant pathogen that is now endemic in South African nurseries [30]. It is one of the most significant pathogens to infect *Pinus* seedlings worldwide [31], with *P. radiata* being highly susceptible [32]. Within the nursery environment, *Pinus* seedlings often succumb to *F. circinatum* infection. Initial symptoms include wilting and discoloration of the growing tip, with death of the root tips and collar rot observed in later stages of infection. Fungal growth on the seedling stem may be visible at an advanced stage of infection [33]. Britz et al. [34] note that *F. circinatum* is the most significant of pathogens infecting *Pinus*, with the fungus now prevalent in *P. radiata* plantations across the Western Cape province of South Africa [31].

### 2.2. Seedling Inoculation

A total of 100 seedlings were randomly sampled from two trays of 3-month old *P. radiata* seedlings (*n* = 196). The seedlings were subsequently divided into two equal classes (*n* = 50) labelled healthy and infected. For the infected class, seedling inoculation followed the PCF Screening Facility Best Operating Practice (Forestry and Agricultural Biotechnology Institute: Pretoria, South Africa) inoculum procedure. This procedure involved first topping the apical buds, followed by placing a 10 μL spore suspension (50,000 spores mL^−1^) of *F. circinatum* isolate (FCC 3579) onto the topped apical buds. Seedlings were kept in a greenhouse for the duration of the study.

### 2.3. Spectroscopic Data Acquisition

**Figure 1:**
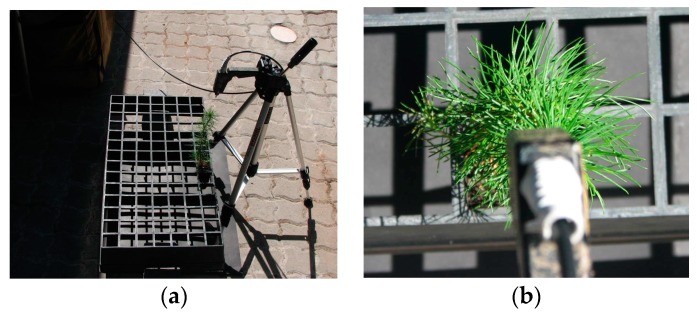
Experimental setup of the spectroradiometer used for spectral data collection (**a**) showing the orientation (nadir view) of the pistol relative to the seedling (**b**).

Spectral data was collected weekly between 10:00 and 15:00 using a FieldSpec^®^ Pro FR Spectroradiometer (Analytical Spectral Devices, Boulder, CO, USA) over a three week period following inoculation. The instrument acquires data in the 350–2500 nm spectral range with a spectral resolution of 3 nm in the visible-near infrared (VIS-NIR) region (350 nm to 1000 nm) and 10 nm in the near infrared-shortwave infrared (NIR-SWIR) region (1000 nm to 2500 nm). Reflectance measurements were calibrated using a Spectralon^®^ white reference panel [35]. Five spectral measurements were captured per seedling using the 23° field-of-view [3,4]. The experimental setup of the spectroradiometer for all data collection is shown in Figure 1. Spectra were later averaged to a single reading per seedling [36]. The spectral data was then pre-processed to remove atmospheric water absorption bands (1350–1460 nm and 1790–1960 nm) [37,38], and noisy bands (2401–2500 nm). 

**Figure 2:**
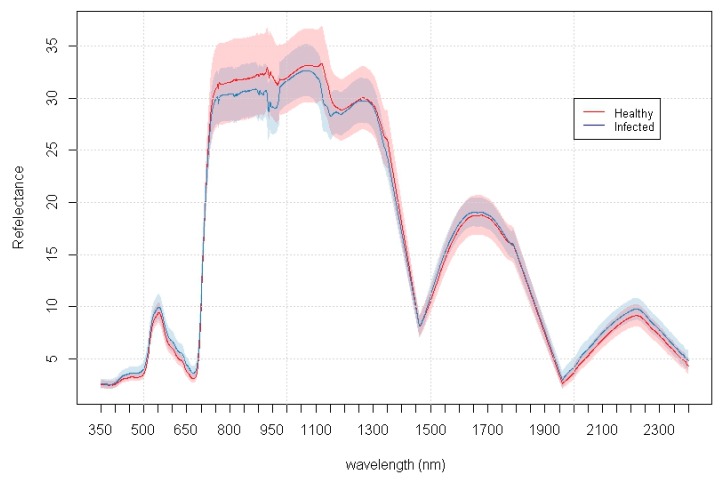
Mean spectral signature of the healthy (*n* = 50) and infected (*n* = 50) classes. The Healthy (sd) and Infected (sd) signatures represent the 1-sigma standard deviation for the healthy (pink shade) and infected (blue shade) signatures respectively.

Figure 2 illustrates the mean spectral signature of the healthy and infected seedlings captured at week one.

### 2.4. Tree-Based Ensembles

#### 2.4.1. Random Forest

The RF algorithm is an extension of bootstrap aggregation of classification and regression trees [12]. The RF algorithm builds models by aggregating large numbers of trees (*ntree*) on bootstrap samples of the original dataset. Trees are maximally grown, i.e., trees are not pruned. To reduce the correlation between trees in the ensemble, the RF algorithm randomly selects a subset of bands (*mtry*) to create the node splits for individual trees in the ensemble. The *mtry* hyperparameter value is equal to the number of bands randomly sampled as candidates for node splitting in each tree. The *mtry* hyperparameter controls the bias variance tradeoff since using fewer bands per node will produce less correlated trees, thereby reducing the overall variance but increasing the bias, as individual trees are now less accurate [15]. The default *mtry* value is equal to the square root of the total number of bands (*p*). The final classification is based on a majority vote of predictions of all trees in the ensemble [39]. Random forest was implemented using the randomForest library [40] in the R statistical software [41]. We used the default *mtry* hyperparameter value (*mtry* = *p*^1/2^) and an *ntree* value of 500 for model building [40].

#### 2.4.2. Oblique Random Forest

The oRF model shares the same ensemble creating process (i.e., bootstrap aggregation and the selection of random variables for node splitting) as RF, but differs in the manner in which the optimal split direction at each node of the tree is created. The original RF implementation uses random coefficients to create optimal splits using a single variable selected from the user defined *mtry* variables whereas oRF uses all the selected *mtry* variables to learn the optimal split direction using a supervised model. Additionally, unlike the original RF implementation, oRF scales (zero mean and unit variance) the variables to enhance model stability [16]. According to [16] models for the node, splits may consider (i) class label information only (for example logistic regression and linear discriminant analysis (LDA)); (ii) data variation (for example principal component analysis); or (iii) an optimum between class label correlation and data (for example ridge regression, partial least squares (PLS), and SVM).

In this study we considered (i) ridge regression; (ii) PLS; and (iii) SVM for multivariate node splitting. Ridge regression aims to improve determination of the regression coefficients and reduce the variance among highly correlated bands by imposing a penalty on the coefficients [42]:

(1) $$RSS\left(\lambda \right)=\overset{n}{\underset{i=1}{\sum }}{\left(y_{i}-{\hat{y}}_{i}\right)}^{2}+\lambda \overset{p}{\underset{j=1}{\sum }}\beta_{j}^{2}$$

where λ controls the shrinkage of the regression coefficients, *n* is the number of samples, *y* is class label, $\hat{y}$ is the regression prediction, *p* is the number of bands, and *β_j_* is the *j*th regression coefficient.

PLS computes a set of weights and loadings for a set of factors that is used to model the variance among the bands and the classes. These weights and loadings are further used to compute the cumulative importance (*B*-value) of each band; the higher the *B*-value, the higher the band importance [43]:

(2) $$B=w{\left(p\prime w\right)}^{-1}q\prime$$

where *B* is the cumulative wavelength importance, *w* is the band weight, *p* is the band loading, and *q* is the class weight.

For a training dataset of *k* classes represented by {*x_i_*,*y_i_*}, *i* = 1, …, *k*, where *x* ∈ **R**^N^ is an N-dimensional space and *y* ∈ {−1,+1} is the class label, SVM seeks to find a separating hyperplane that maximizes the perpendicular distance between the healthy and infected classes by solving the constrained optimization problem [10]:

(3) $$\underset{w,b}{\min}\frac{1}{2}{∥w∥}^{2}$$

where w is a vector that determines the orientation of the separating hyperplane, and *b* is a scalar that determines the offset of the hyperplane from the origin.

For all models, the regularization parameters were optimized using the out-of-bag (OOB) samples at each node [37]. Oblique random forest was implemented using the obliqueRF library [44] in the R statistical software [34]. We used the default hyperparameter values of *mtry* (i.e., the square root of the total number of bands) and *ntree* value of 300 for model building [16].

#### 2.4.3. Rotation Forest

Rotation forest is a tree based ensemble approach [38] that uses DT as the base learner. It is similar to RF with respect to training independent trees, but differs by using a different subset of extracted features to train each tree. The key principle underpinning rotation forest is the use of PCA to first transform the original feature space to a new rotated feature space and subsequently undertake feature extraction for each base classifier [18]. Feature extraction is applied to subsets of bands, with all principal components then used for training each DT. Random partitioning of the feature set leads to greater diversity of the bootstrap samples. Similar to RF, the final classification result is based on a majority vote of the combined DT [45]. Rotation forest was implemented in the R statistical software [34], using *ntree* = 100 and the default hyperparameter values of *mtry* (i.e., the square root of the total number for bands) for building our models. We used *ntree* = 100 given that using *ntree* = 10 [18] did not provide valuable results (not shown).

### 2.5. Spectral Resampling

In this study, we used spectral resampling to reduce data dimensionality, and subsequently test the effect of a reduced dimensionality on classification accuracy. Two studies [46,47] used a stepwise merging approach, which involved summation of the full width at half maximum (FWHM) values of adjacent bands, to resample HyMap spectra. [13,48] applied user-defined bandwidths (equivalent to FWHM) fit to a Gaussian (normal distribution) model to resample spectral measurements to HyMap spectra. One study [49] used the mean of contiguous spectral bands to spectral resample AISA Eagle bands ranging from 4.6 nm to 36.8 nm in increments of 4.6 nm. In this study we incrementally resampled the original bands (*n* = 1769) using user-defined waveband centers, based on the mean of adjacent bands. Subsets of bands were created by binning (resampling) bands into specified wavelength ranges, i.e., from 2 nm to 176 nm. Resampling of the hyperspectral bands was performed using the pavo library [50] in the R statistical software [41]. The resulting eight subsets ranged in size from *n* = 884 to *n* = 10 bands that were then used to test the robustness of the ensemble classifiers used in this study.

### 2.6. Classification Accuracy

An independent test dataset (i.e., captured during week two) was used for assessing classification accuracy. This provided an independent estimate of model accuracy. All algorithms were trained using the spectral measurements obtained during week one and subsequently tested using the spectral measurements collected during week two of the experiment. Classification accuracy was then evaluated using overall accuracy derived from a confusion matrix [51]. Additionally, we used a discrete multivariate technique called Kappa analysis to assess classification accuracy. A KHAT statistic [52] provides a measure of agreement between actual (“observed”) agreement and chance (“expected”) agreement:

(4) $$\hat{K}=\frac{p_{o}-p_{c}}{1-p_{c}}$$

where *p_o_* is the actual agreement and *p_c_* is the expected agreement. To provide a more robust measure of model generalization, models were replicated (*n* = 100) [53] and descriptive statistics (mean accuracy and standard deviation) computed.

## 3. Results

**Figure 3:**
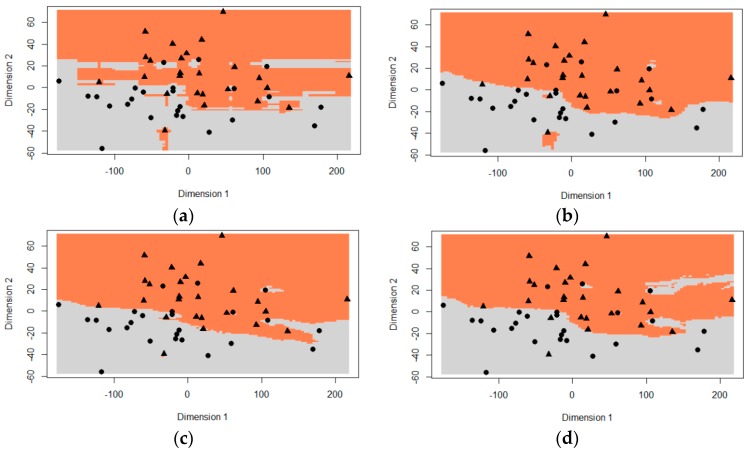
Visualization of the decision boundary for (**a**) RF; (**b**) oRFridge; (**c**) oRFpls; and (**d**) oRFsvm. The margin between the gray and coral areas represents the decision boundary learned. The dots and triangles represent the two classes, i.e., healthy and infected. RF = random forest; rotF = rotation forest; oRFridge = oblique random forest using ridge regression as splitting model; oRFpls = oblique random forest using PLS as splitting model; oRFsvm = oblique random forest using SVM as splitting model.

To better understand the difference in behavior of the RF and oRF models, we examined the topology of the decision boundary learned by each ensemble classifier (Figure 3). The decision boundary was modelled using the first two principal components extracted from a principal components analysis of the original hyperspectral dataset (*n* = 1769). Figure 3a clearly illustrates the staircase or box-like decision boundary generated by univariate orthogonal splits, as used by RF [16,54]. For the oRF ensembles (Figure 3b–d) however, the smoother decision boundary is reminiscent of multiple rotated trees using random multivariate splits [16].

**Figure 4:**
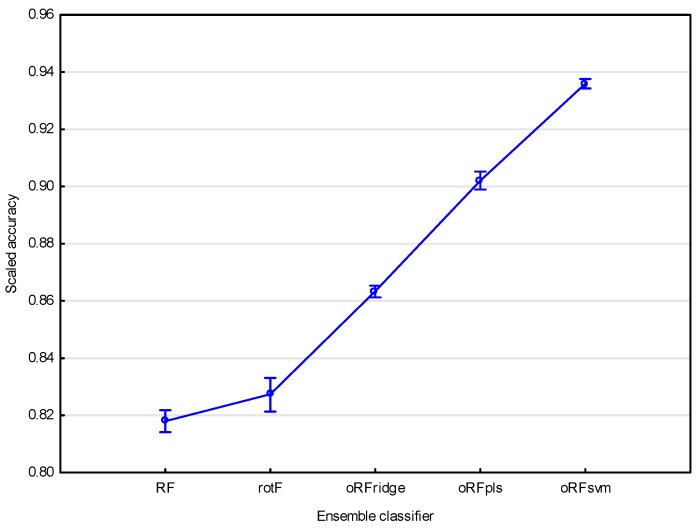
Mean classification accuracies for all tree-based algorithms (RF = random forest; rotF = rotation forest; oRFridge = oblique random forest using ridge regression as splitting model; oRFpls = oblique random forest using PLS as splitting model; oRFsvm = oblique random forest using SVM as splitting model) considered in this study. The scaled accuracy is the classification accuracy represented on a scale ranging from zero to one. Vertical bars denote 0.95 confidence intervals.

Figure 4 shows the resulting mean classification accuracies obtained for the five ensemble classifiers using all bands (*n* = 1769) based on 100 model runs. For all ensembles, the mean model accuracy was above 80% (KHAT values ranged from 0.61 ± 0.16 to 0.87 ± 0.02). The oRFsvm model produced the highest mean classification accuracy of 93.59% ± 0.85%. In comparison, the traditional RF model yielded the lowest mean classification accuracy of 81.8% ± 1.82%. Rotation forest (rotF) yielded a similar accuracy of 82.73% ± 3.06% when compared with RF, but has a higher variability of accuracy values denoted by the wider confidence interval.

**Figure 5:**
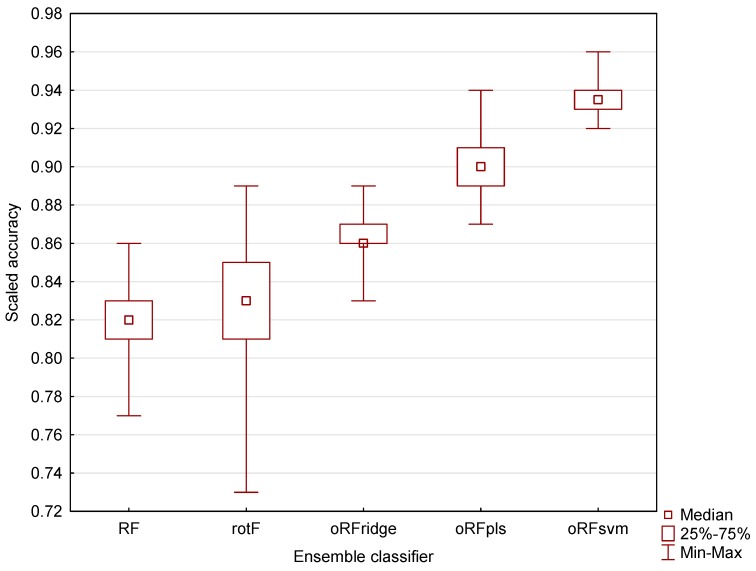
The distribution of the classification accuracy based on the test dataset for all tree-based algorithms (RF = random forest; rotF = rotation forest; oRFridge = oblique random forest using ridge regression as splitting model; oRFpls = oblique random forest using PLS as splitting model; oRFsvm = oblique random forest using SVM as splitting model) considered in this study. Each boxplot represents the results obtained from 100 repetitions and all bands (*n* = 1769). The scaled accuracy is the classification accuracy represented on a scale ranging from zero to one.

It is evident from Figure 5 that the oRFsvm ensemble also has the smallest range of accuracy values between the upper and lower quartiles. This indicates higher classification results and better generalization ability when compared with the other ensembles. Conversely, the rotF model has the largest range of accuracy values between the upper and lower quartiles. This indicates lower generalization ability.

To determine if the classification accuracies obtained using the five tree-based ensemble classifiers were statistically different, we performed a one-way ANOVA followed by Fishers LSD test [55] with bootstrapping [56]. The results showed that there was no significant difference between the accuracies obtained for the RF and rotF models at *p* = 0.05. However, there was a significant difference between the accuracies obtained for the three oRF models, i.e., oRFridge, oRFpls, and oRFsvm. Additionally, there was a significant difference between the RF model accuracy and the oRFridge, oRFpls, and oRFsvm model accuracy, as well as between the rotF model accuracy and the oRFridge, oRFpls, and oRFsvm model accuracy. Figure 5 indicates that the oRFridge, oRFpls, and oRFsvm models produced significantly higher mean accuracies (ranging between 86% and 94%) compared with RF and rotF models that produced significantly lower, and statistically similar, accuracies (ranging between 80% and 84%).

**Figure 6:**
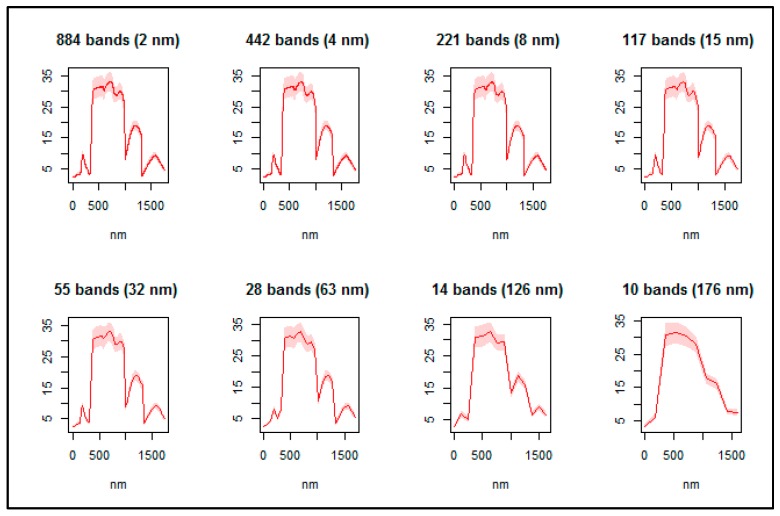
Resampling of the original hyperspectral dataset. Subsets of bands ranged in size from *n* = 884 (spectral resampling to 2 nm) to *n* = 10 (spectral resampling to 176 nm). The *X*-axis represents the wavelength (nm) of the resampled bands whereas the *Y*-axis represents the reflectance (%).

**Figure 7:**
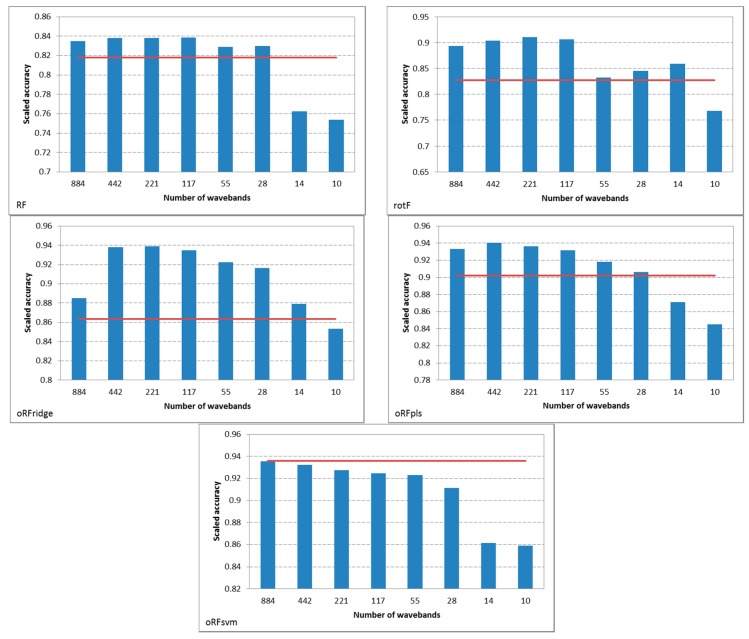
Comparison of the mean accuracies obtained using all bands and using the resampled bands for the five ensemble classifiers (RF = random forest; rotF = rotation forest; oRFridge = oblique random forest using ridge regression as splitting model; oRFpls = oblique random forest using PLS as splitting model; oRFsvm = oblique random forest using SVM as splitting model). The red line indicates the mean accuracy obtained using all the original bands (*n* = 1769) whereas the blue bars indicate the mean accuracies for the respective resampled subsets.

Figure 6 shows the result of spectral resampling of the original hyperspectral dataset (*n* = 1769). Resampling of the hyperspectral bands resulted in subsets of bands ranging in size from *n* = 884 (resampled to 2 nm) to *n* = 10 (resampled to 176 nm). These subsets were used to generate models using each of the five ensemble classifiers. The results illustrated in Figure 7 show that for all ensembles, except oRFridge, the mean classification accuracy remained stable when using bands resampled to 2 nm ranging up to 63 nm. However, bands resampled to 126 nm and 176 nm show a significant decrease in mean classification accuracy for all ensembles considered in this study. The oRFsvm ensemble provided the most consistent accuracies across all resampled bands and is thus shown to be the most robust of all the ensembles considered in this study.

**Figure 8:**
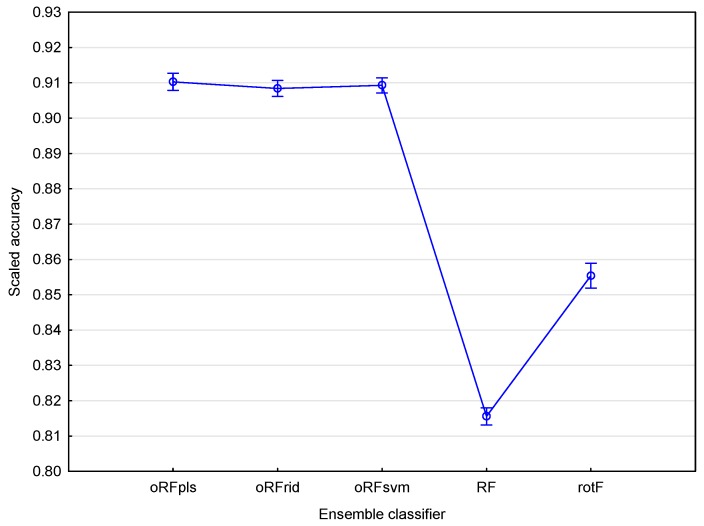
Mean classification accuracies using resampled hyperspectral bands (*n* = 800) for each of the tree-based algorithms (RF = random forest; rotF = rotation forest; oRFridge = oblique random forest with ridge regression as splitting model; oRFpls = oblique random forest with PLS as splitting model; oRFsvm = oblique random forest with SVM as splitting model) considered in this study. The scaled accuracy is the classification accuracy represented on a scale ranging from zero to one. Vertical bars denote 0.95 confidence intervals.

We again performed a one-way ANOVA followed by Fishers LSD test [55] with bootstrapping [56] to determine if the classification accuracies of all the ensemble models obtained using the spectral resampled bands were statistically different. The results show that there was no significant difference in accuracy between the three oRF models, i.e., oRFridge, oRFpls, and oRFsvm, at *p* = 0.05. This is contrary to the results obtained when using all hyperspectral bands. The results also indicated that the RF and rotF model accuracies were significantly different from each other as well as from the oRFridge, oRFpls, and oRFsvm model accuracies. It is clear from Figure 8 that the oRFridge, oRFpls, and oRFsvm models produced similar accuracies (ranging between 90% and 92%) compared with the RF and rotF models which have significantly lower mean accuracies.

Table 1 summarizes the highest and lowest mean classification accuracies (and associated spectral resampled bands) for all the ensemble classifiers considered in this study. Overall results indicate that the three oRF ensembles, i.e., oRFridge, oRFpls, and oRFsvm, produced the highest mean classification accuracies. Additionally, the oRFridge model had the lowest standard deviation of 0.48 when using bands (*n* = 221) resampled to 8 nm. In comparison, RF produced a highest mean classification accuracy of only 84% ± 0.60% using bands (*n* = 117) resampled to 15 nm. For all ensembles, classification using a very coarse spectral resolution, that is spectral resampling to 176 nm (*n* = 10), yielded the lowest mean classification accuracy.

Comparing the results in Table 1 with the mean classification accuracies obtained using all bands (*n* = 1769), it is evident that spectral resampling resulted in an overall increase in classification accuracy. For example, for rotation forest, the highest mean classification accuracy achieved was 91% ± 0.85%, using bands (*n* = 221) resampled to 8 nm compared with 83% ± 3.06% using all bands. This is equivalent to an increase of more than 8% in classification accuracy. The only exception, in which there was no change in classification accuracy, was for oRFsvm with a highest mean classification accuracy of 94% ± 0.77% using the resampled bands compared with 94% ± 0.85% using all bands.

## 4. Discussion

Tree-based ensemble classifiers are widely used for the classification of high dimensional data (see for example [3,4,5,6]). Their popularity is driven by the basic premise that using many weak classifiers should yield better classification accuracy than a single classifier [57]. In this study, we compared five tree-based ensemble classifiers, i.e., random forest (RF), rotation forest (rotF), oRF using ridge regression as the splitting model (oRFridge), oRF using PLS as the splitting model (oRFpls), and oRF using SVM as the splitting model (oRFsvm). We specifically examined the effect of spectral resolution on the ensemble’s ability to classify healthy and infected *P. radiata* seedlings using high dimensional spectral data. The following sections discuss the experimental results in more detail.

### 4.1. Classification Using All Bands

Random forest has become a popular ensemble classifier for the analysis of hyperspectral data, given that it is relatively robust to outliers and noise and is not prone to over-fitting [58]. Our analysis shows that RF was generally outperformed by the other tree-based ensembles considered in this study. This indicates that RF may not be the optimal ensemble classifier for the classification of spectroscopic data. When using all bands (*n* = 1769) the RF ensemble only marginally outperformed rotation forest with a mean classification accuracy of 82% ± 1.82% for RF compared with 79% ± 3.06% for rotation forest. More importantly, RF was significantly outperformed by oRFridge (86% ± 1.06%), oRFpls (90% ± 1.66%), and oRFsvm (94% ± 0.85%).

Contrary to previous studies (for example [18,21,59]) that have demonstrated the superior performance of rotation forest compared with RF, this study shows that rotation forest produced the lowest overall classification accuracies. Rotation forest was the least robust of all the ensemble classifiers, yielding variable classification accuracies ranging from a minimum of 73% to a maximum of 89% with a standard deviation of 3.06%.

However, the results of this study compare favorably with those of [14,16]. For example, Do et al. [14] tested the performance of RF, SVM, and oRF. The key finding of their study was that oRF outperformed both RF and SVM by an average of 3.57% and 6.35% respectively. Our results show that oRF (using SVM as the splitting rule) outperformed RF by an average of 12%. Menze et al. [16] also showed that for the classification of high dimensional spectral data, the RF ensemble was outperformed by the oRF ensembles, with oRFridge yielding the best classification result. Our analyses further indicate that although oRFridge outperformed RF, oRFridge was outperformed by both oRFpls and oRFsvm, with the oRFsvm ensemble providing the best classification accuracy when using the entire hyperspectral dataset. We attribute the stable results of oRFsvm to the ability of SVM to effectively handle ill-posed problems, i.e., classification of a high dimensional feature space with limited training samples, coupled with its higher generalization ability [60]. Of note is that only a limited number of studies have investigated the use of oRF for the analysis of high dimensional data; see for example [14,16,61]. Additionally, the results of our study highlight the potential to use oRF in a binary application.

### 4.2. The Effect of Spectral Resampling on Classifier Performance

In this study, a total of 100 samples was used, i.e., healthy (*n* = 50) and infected (*n* = 50). All models were constructed using a decreasing number of bands (*p*) while maintaining the number of samples (*n*) constant. Models constructed from a larger number of samples compared with the number of bands (*n* < *p*) generally achieved the highest accuracy. This is evident from Figure 7, where the highest accuracies are obtained using bands spectral resampled to 2 nm, 4 nm, 8 nm, and 15 nm. A similar result is observed for models constructed with an equivalent number of samples and bands (*n* ≈ *p*); evident using bands spectral resampled to 32 nm to 63 nm. However, models constructed with a lower number of bands compared with the number of samples (*n* > *p*) showed the lowest classification performance. These results are evident using bands spectral resampled to 126 nm to 176 nm. This trend was also observed by [47,49] who found that models constructed from a lower number of bands yielded the lowest accuracies.

Spectral resampling of the hyperspectral bands produced mixed results with respect to the ensemble model employed. For example, from an evaluation of the mean classification accuracy obtained for RF, rotF, and oRFpls using the original bands compared with using the spectral resampled bands, it is evident that improved classification performance was achieved using the spectral resampled bands. For oRFridge and oRFpls, using spectral resampled bands yielded a significant increase in the classification performance. However, for oRFsvm, using spectral resampled bands did not yield any significant improvement in the mean classification accuracy. Several authors (see for example [62,63,64]) have found that the performance of the linear SVM is not significantly influenced by a reduced dimensionality. The robustness of SVM has already been illustrated using oRFsvm for the classification using all bands (Section 4.1). Similar results were demonstrated by [49] using the SVM, Gaussian maximum likelihood with leave-one-out-covariance estimator (GML-LOOC), and LDA classifiers. The authors noted that the SVM classifier yielded the highest Kappa accuracies, and remained stable across all spectral resampled subsets. Kappa accuracies were generally lower for the GM-LOOC and LDA classifiers.

Overall, our results reaffirm the findings of previous research [3,4], demonstrating that decreasing the data dimensionality leads to improved overall classification accuracy, and that a lower dimensional dataset can be used to efficiently discriminate healthy and infected seedlings. In this study, all ensemble classifiers displayed a similar trend in classification performance with the resampled datasets, i.e., classification accuracy remained stable at lower FWHM values and decreased at higher FWHM values. A similar trend was observed by [47,49]. Although lower accuracies were obtained at a spectral resolution of 126 nm and 176 nm, the results indicate that it is still possible to discriminate the two classes (healthy and infected). For example, for both RF and rotF, classification accuracy was above 75% using bands resampled to 176 nm. In the case of oRFridge, oRFpls, and oRFsvm, classification accuracy was above 84% using bands resampled to 176 nm.

### 4.3. Robustness of the Oblique Forest Ensembles

To model asymptomatic stress in *P. radiata* seedlings associated with *Fusarium circinatum* infection we evaluated the use of random forest ensembles including rotation forest and oblique random forest. Previous studies (for example [14,16]) have demonstrated the superior performance of oblique forest ensembles compared with other classifiers such as RF, classification and regression trees (CART), and SVM. The use of oblique random forest was found to be particularly suitable for the processing of high dimensional spectral data.

As previously indicated, the staircase or box-like decision boundary generated by univariate splits, as is the case with CART and RF, may not be optimal for the classification of highly correlated data, such as high dimensional spectroscopic data [16]. Consequently, learners that comprise multivariate DT via generation of oblique decision boundaries would be more suited to analyzing high dimensional, highly correlated hyperspectral data. The results obtained in this study clearly confirm this notion. In this study, the traditional RF ensemble constructed from univariate DT was outperformed by all three oRF ensembles as well as the rotation forest ensemble, which are constructed from multivariate DT. Additionally, the use of an algorithm—in this study we used ridge regression, PLS, and SVM—to estimate the splitting rule for the oRF ensembles likely contributed to the improved performance of the oRF ensemble and consequently the high classification accuracies. Freedl and Brodley [65] showed that multivariate DT incorporating splitting rules produced significantly higher classification accuracies compared with univariate DT and Bayesian classifiers. Similarly, Pal and Mather [66] showed that multivariate DT produced comparatively high classification accuracies compared with univariate DT, artificial neural networks, and Bayesian classifiers.

The classification results further indicate that the performance of the oRF ensembles is not significantly affected by the multicollinearity, albeit the fact that higher classification accuracies were obtained when a lower dimensionality, i.e., spectral resolution was used. In this study we systematically reduced the dataset size by spectral resampling (binning) of the original dataset (*n* = 1769) into discrete subsets of wavebands. The results of [13,46,47,48,49] illustrate that reducing the input data dimensionality results in improved classification performance. This notion is reinforced by the results achieved in this study using the oRF ensembles to classify high dimensional spectroscopic data. We have demonstrated that a subset of bands, generated by spectral resampling of the original dataset (*n* = 1769), achieves accuracies above 90%, when an oblique node-splitting model is used.

The results of this study thus demonstrate the potential for operationalization of the oblique ensemble model for the asymptomatic detection of *Fusarium circinatum* infection in *Pinus radiata* seedlings within a nursery environment.

## 5. Conclusions

This study aimed to evaluate the performance of various ensemble classifiers for the analysis of high dimensional spectral data. Additionally, the study tested the robustness of these ensembles to reduced data dimensionality and sample size. Some important conclusions from this study are, firstly, that rotation forest and oRF may be more suitable than RF for the analysis of high dimensional spectral data. Secondly, rotation forest is sensitive to both dimensionality and sample size, and produces less robust models compared with RF and oRF. Thirdly, the oRF ensemble using varied splitting models is most robust and yields better classification results compared with rotation forest and RF. Finally, the methods employed in this study require further investigation to evaluate their operational potential.

## Figures and Tables

**Table 1:** Spectral resampled wavelengths and the associated classification results using the five ensemble classifiers (RF = random forest; rotF = rotation forest; oRFridge = oblique random forest using ridge regression as splitting model; oRFpls = oblique random forest using PLS as splitting model; oRFsvm = oblique random forest using SVM as splitting model). KHAT values are indicated in parentheses.

Ensemble Classifier	Highest Accuracy (%)	Resampled Bands (nm)	Resampled Bands (*n*)	Lowest Accuracy (%)	Resampled Bands (nm)	Resampled Bands (*n*)
RF	84 ± 0.60 (0.68 ± 0.01)	15	117	75 ± 1.35 (0.51 ± 0.03)	176	10
rotF	91 ± 0.85 (0.80 ± 0.04)	8	221	77 ± 1.24 (0.55 ± 0.07)	176	10
oRFridge	94 ± 0.48 (0.88 ± 0.01)	8	221	85 ± 1.75 (0.71 ± 0.03)	176	10
oRFpls	94 ± 0.75 (0.88 ± 0.02)	4	442	85 ± 1.28 (0.69 ± 0.03)	176	10
oRFsvm	94 ± 0.77 (0.87 ± 0.02)	2	884	86 ± 1.20 (0.72 ± 0.03)	176	10
